# The relationship between the structural changes in the cervical spinal cord and sensorimotor function of children with thoracolumbar spinal cord injury (TLSCI)

**DOI:** 10.1038/s41393-024-01000-w

**Published:** 2024-06-01

**Authors:** Qunya Qi, Ling Wang, Beining Yang, Yulong Jia, Yu Wang, Haotian Xin, Weimin Zheng, Xin Chen, Qian Chen, Fang Li, Jubao Du, Jie Lu, Nan Chen

**Affiliations:** 1 Department of Radiology and Nuclear Medicine, Xuanwu Hospital, Capital Medical University, 100053 Beijing, China; 2 Beijing Key Laboratory of Magnetic Resonance Imaging and Brain Informatics, 100053 Beijing, China; 3 Department of Radiology, Beijing Chaoyang Hospital, Capital Medical University, 100020 Beijing, China; 4 Department of Radiology, Beijing Friendship Hospital, Capital Medical University, 100050 Beijing, China; 5 Department of Rehabilitation Medicine, Xuanwu Hospital, Capital Medical University, 100053 Beijing, China

**Keywords:** Diagnostic markers, Biomarkers

## Abstract

**Study design:**

Cross-sectional study.

**Objectives:**

To study the relationship between the structural changes in the cervical spinal cord (C2/3 level) and the sensorimotor function of children with traumatic thoracolumbar spinal cord injury (TLSCI) and to discover objective imaging biomarkers to evaluate its functional status.

**Setting:**

Xuanwu Hospital, Capital Medical University, China; Beijing Key Laboratory of Magnetic Resonance Imaging and Brain Informatics, China.

**Methods:**

30 children (age range 5–13 years) with TLSCI and 11 typically developing (TD) children (age range 6–12 years) were recruited in this study. Based on whether there is preserved motor function below the neurological level of injury (NLI), the children with TLSCI are divided into the AIS A/B group (motor complete) and the AIS C/D group (motor incomplete). A Siemens Verio 3.0 T MR scanner was used to acquire 3D high-resolution anatomic scans covering the head and upper cervical spinal cord. Morphologic parameters of the spinal cord at the C2/3 level, including cross-sectional area (CSA), anterior-posterior width (APW), and left-right width (LRW) were obtained using the spinal cord toolbox (SCT; https://www.nitrc.org/projects/sct). Correlation analyses were performed to compare the morphologic spinal cord parameters and clinical scores determined by the International Standard for Neurological Classification of Spinal Cord Injuries (ISNCSCI) examination.

**Results:**

CSA and LRW in the AIS A/B group were significantly lower than those in the TD group and the AIS C/D group. LRW was the most sensitive imaging biomarker to differentiate the AIS A/B group from the AIS C/D group. Both CSA and APW were positively correlated with ISNCSCI sensory scores.

**Conclusions:**

Quantitative measurement of the morphologic spinal cord parameters of the cervical spinal cord can be used as an objective imaging biomarker to evaluate the neurological function of children with TLSCI. Cervical spinal cord atrophy in children after TLSCI was correlated with clinical grading; CSA and APW can reflect sensory function. Meanwhile, LRW has the potential to be an objective imaging biomarker for evaluating motor function preservation.

## Introduction

Spinal cord injury (SCI) can lead to permanent motor and sensory deficits [1, 2]. When SCI occurs in childhood, it may cause severe physiological and psychological consequences [3, 4]. A series of pathophysiological processes are started at the site of damage immediately after trauma, including the destruction of neurons and oligodendrocytes, disruption of the axonal network, hemorrhage, and growth factor dysregulation [1, 5–7]. The primary injury initiates a cascade of secondary pathological processes that spread above and below the site of injury and also affect the brain [1, 2, 6–8].

Numerous studies have confirmed that rapid and progressive spinal cord atrophy will occur after SCI, which may be the cumulative result of multiple pathophysiological processes following SCI [5, 6]. At present, the morphological changes of the cervical spinal cord have become an important indicator for judging spinal cord atrophy and functional status [5, 9–12]. Cross-sectional cervical spinal cord area (cSC CSA) is a common MRI measurement used to assess spinal cord atrophy after SCI. Changes in cSC CSA are strongly associated with functional outcomes [5, 6, 9–11]. The smaller the cSC CSA of people with SCI, the worse their motor and sensory function and functional independence [9–11]. One study found that the anterior-posterior width (APW) correlated well with sensory scores, while the left-right width (LRW) correlated with motor scores [11]. This may be because the sensory pathways are mainly located in the dorsal and ventral columns of the spinal cord, while the motor sensory pathways are mainly located in the lateral columns of the spinal cord [11, 13, 14]. Another study of acute SCI found that APW at baseline was associated with lower limb motor scores at 2 months after injury [6]. Therefore, quantitative assessment of spinal cord atrophy after SCI may provide additional helpful biomarkers for injury assessment and the prediction of neurologic recovery [12]. At present, these studies mainly focused on adult SCI, and there have been no reports on the morphology of the cervical spinal cord after SCI in children. Therefore, the morphological changes of the cervical spinal cord after SCI in children and their relationship with clinical function were still unclear. Meanwhile, the International Standard for the Neurological Classification of Spinal Cord Injury (ISNCSCI) was the most commonly used classification system for SCIs [4, 15, 16]. However, the ISNCSCI has no practical application for children younger than 6 years of age, and the ISNCSCI may not be able to accurately assess the neurological consequences in children with SCI [4, 15, 17]. Therefore, it is important to seek relatively robust and objective imaging biomarkers to assess the functional status of children with SCI.

Unlike adults, the area of the spinal cord in children increases with age [18, 19]. It has been shown that the spinal cord at the C2/C3 level in TD children grows faster than at other vertebral levels [18]. Nevertheless, there were no reports on structural changes in the spinal cord at the C2/3 level above the injury level in children after SCI, and the relationship between functional outcomes and cSC CSA in children with SCI has not been studied. Therefore, this study aimed to investigate whether the distal cervical spinal cord (C2/3 level) structure alterations after thoracolumbar SCI (TLSCI) in children and to explore the correlation between structural changes and motor/sensory function, in an attempt to find objective imaging biomarkers to help clinically assess the function of children with SCI.

## Methods

### Participants

A total of 41 female participants were recruited for this study, including 30 children with traumatic TLSCI (age range 5–13 years, time since injury ≥2 months, thoracolumbar injury; mean age 8.4 years); and 11 typically developing (TD) children (age range 6–12 years; mean age 8.6 years). The study received approval from the ethics committee of Xuanwu Hospital and the Helsinki Declaration, and the parents or guardians of participating children provided written informed consent. The demographic data of children with TLSCI are shown in Table 1. According to the American Spinal Injury Association Impairment Scale (AIS) grade, the children with TLSCI were divided into AIS A/B group (motor complete, including 14 AIS A, 3 AIS B; age range 6–12 years; mean age 8.5 years); and AIS C/D group (motor incomplete; total 13; 5 AIS C, 8 AIS D; age range 5–13 years; mean age 8.2 years). All the children with TLSCI underwent a complete ISNCSCI examination by one experienced rehabilitation physician, including light touch sensory scores (LTSS) and pinprick sensory scores (PPSS) of all segments from C2 to S4–5 on the right and left sides and motor testing for the five major muscle functions of the upper and lower extremities [20].

**Table 1:** Demographic data of children with TLSCI.

Participant ID	Age at enrollment (years)	Gender	Etiology	Level of lesion	Initial AIS grade	AIS grade at inclusion	Time since injury (months)	Motor scores (0–100)	PPSS (0–112)	LTSS (0–112)
1	9	F	Backbend	T8	A	A	12	50	64	64
2	9	F	Backbend	T4-T9	A	A	14	50	64	64
3	6	F	Backbend	T9-L1	A	A	14	50	64	64
4	7	F	Backbend	T4-T5	A	A	21	50	64	64
5	9	F	Backbend	T4-T7	A	A	21	50	72	72
6	6	F	Backbend	T9	A	A	15	50	68	68
7	6	F	Backbend	T4	A	A	12	50	52	52
8	9	F	Backbend	T8-T12	A	A	18	50	68	68
9	7	F	Backbend	T9-T11	A	A	30	50	64	64
10	12	F	Backbend	T10	A	A	24	50	64	64
11	11	F	Backbend	T9-T10	A	A	48	50	64	64
12	11	F	Backbend	T7-T10	A	A	36	50	64	64
13	7	F	Backbend	T10	A	A	24	50	64	64
14	7	F	Backbend	T8–T12	A	A	29	50	60	60
15	11	F	Backbend	T7-L1	B	B	3	50	72	72
16	9	F	Backbend	T10-T11	B	B	2	50	88	88
17	10	F	Backbend	T9-L1	B	B	3	50	84	58
18	5	F	Fall	T10-T11	B	C	27	80	94	94
19	8	F	Fall	T10	C	C	5	88	102	102
20	8	F	Backbend	T6	B	C	2	74	88	88
21	7	F	Backbend	T1-L1	C	C	7	60	70	70
22	10	F	Fall	T11	C	C	11	70	70	70
23	7	F	Sport injury	T3	D	D	22	90	84	84
24	13	F	Backbend	T9-T12	B	D	87	76	84	84
25	8	F	Backbend	T12-L1	D	D	19	90	94	94
26	13	F	Bleed	T5-T7	C	D	21	75	84	84
27	5	F	Stabbed	T8-T12	A	D	38	90	84	84
28	10	F	Backbend	T10-T12	C	D	11	90	86	86
29	7	F	Backbend	T5-L2	B	D	13	75	94	94
30	6	F	Backbend	T4-T7	C	D	4	90	110	110

The level of lesion refers to the MRI level.

*TLSCI* thoracolumbar spinal cord injury, *F* female, *AIS* American Spinal Injury Association Impairment Scale, *PPSS* Pinprick Sensory Scores, *LTSS* Light touch Sensory Scores.

TD children met the following inclusion requirements: 1) Conventional MRI of the brain showed no abnormalities; 2) No history of drug addiction, psychological disorders, or inherited family diseases; 3) MRI images met study requirements; 4) No contraindications to MRI.

All participants with SCI satisfied met the following requirements: 1) Children with TLSCI; 2) Conventional head MRI showed there were no abnormalities in the brain; 3) More than 2 months after injury; 4) No history of drug addiction, psychological disorders, or traumatic brain injury; 5) No contraindications to MRI; 6) MRI images met study requirements.

Participants who had the following conditions will be excluded from this study: 1) They experienced traumatic brain injury；2) They were unable to tolerate MRI scans; 3) They had any history of neuropsychiatric disorders; 4) Their MRI images’ quality was poor or had artifacts.

### Data acquisition

All MRI images were acquired using a 3.0 T system (Trio Tim, Siemens, Erlangen, Germany) with a 12-channel phased-array head and neck joint coil. All the participants underwent sagittal T1-weighted 3D magnetization-prepared rapid gradient-echo (MPRAGE) sequence scan with the following parameters: repetition time = 1800 ms, echo time = 2.1 ms, inversion time = 1100 ms, flip angle = 9°, voxel size 1 × 1 × 1 mm^3^, field of view (FOV) = 265 × 265 mm^2^, slice thickness = 1 mm, number of slices = 192, acquisition time = 6 min 59 s. The sequence was a standard protocol for the brain, the FOV covered the entire head and upper cervical spinal cord.

### MRI data processing

**Fig. 1:**
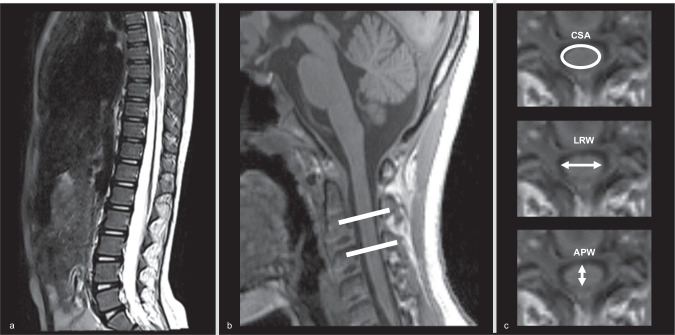
Measurement of morphologic parameters of the cervical spinal cord. **a** A participant with ID 3 suffered TLSCI due to backbend. A sagittal T2-weighted image taken 14 months post-injury shows atrophy of the spinal cord below the T9 to L1 level. **b** 3D T1-MPRAGE midsagittal image of the same participant. The white line shows the assessed location of the spinal cord. **c** Extract the morphologic spinal cord parameters at this location, including spinal cord CSA, APW, and LRW. TLSCI thoracolumbar spinal cord injury, MPRAGE magnetization-prepared rapid gradient-echo, CSA cross-sectional area, APW anterior-posterior width, LRW left-right width.

Spinal Cord Toolbox (SCT; https://www.nitrc.org/projects/sct) was used to process 3D T1-MPRAGE data [21]. The processing steps were as follows: 1) The spinal cord was first segmented using the “sct_propseg” provided by the SCT. “sct_propseg” can be used for pediatric participants. 2) Next, the C2/3 intervertebral discs were identified and the spinal vertebral levels were labeled using “sct_label_vertebrae”. 3) Finally, the mean values of spinal cord morphologic parameters between C2 and C3 were extracted, including spinal cord CSA, anteroposterior width (APW), and left-right width (LRW) (Fig. 1). All of the above steps were manually qualified control, and if automatic division fails, manual labeling will be performed to ensure the accuracy of the spinal cord segmentation and vertebral level labeling. The processing steps were similar to a previous study [20].

### Statistical analysis

SPSS (version 25.0, Armonk, New York, U.S.) software was used to conduct the statistical analysis. One-way Analysis of variance (ANOVA) was used to assess the differences in age and C2/3 spinal cord morphological parameters among the three groups, and the mean time since the injury of the AIS A/B group and AIS C/D group was analyzed using Mann-Whitney U test. Pearson linear correlation was used to assess the correlation between the morphological spinal cord parameters and the ISNCSCI scores in children with TLSCI. Receiver operating characteristic (ROC) analysis was performed on the children with TLSCI, and the specificity and sensitivity of imaging biomarkers to distinguish the two groups of TLSCI were calculated. Multiple linear regression was used to analyze the effect of age, time since injury, and clinical grading on the cervical spinal cord structure. *P* < 0.05 was considered statistically significant.

## Results

### Clinical and demographic characteristics

There was no statistically significant difference in age among the TD group, AIS A/B group, and AIS C/D group (*p* = 0.902). There was no statistically significant difference in the average time since injury between the AIS A/B group and the AIS C/D group (months; U = 96.5, *P* = 0.536).

### Morphologic spinal cord parameters at C2/3 level

**Fig. 2:**
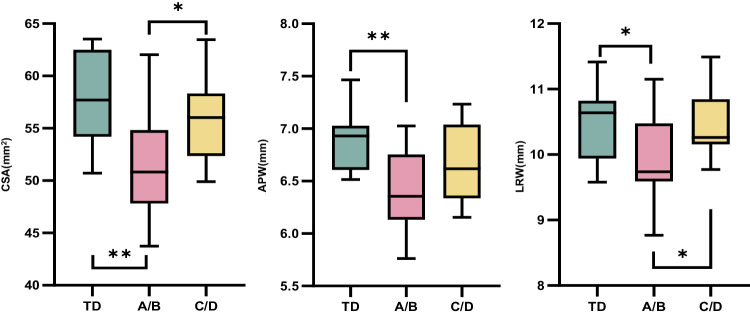
Results of one-way ANOVA of morphologic spinal cord parameters at C2/3 level in the three groups. The AIS A/B group was significantly lower than those in the TD group (CSA: *p* = 0.002; APW: *p* = 0.001; LRW: *p* = 0.029), while CSA and LRW were significantly smaller than AIS C/D (CSA: *p* = 0.014; LRW: *p* = 0.026). ANOVA One-way Analysis of variance, CSA cross-sectional area, APW anterior-posterior width, LRW left-right width, TD typically developing, AIS American Spinal Injury Association Impairment Scale. *p ≤ 0.05; **p ≤ 0.01.

**Fig. 3:**
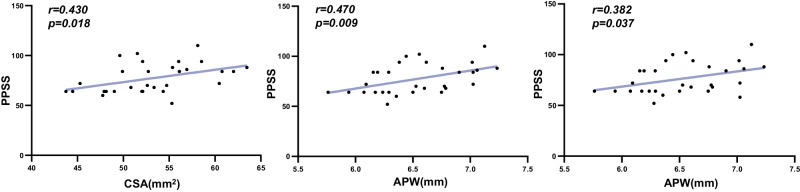
Associations between CSA, APW, LRW, and ISNCSCI Scores. CSA and PPSS were positively correlated (*r* = 0.430, *p* = 0.018). APW was positively correlated with PPSS (*r* = 0.470, *p* = 0.009) and LTSS (*r* = 0.382, *p* = 0.037). CSA cross-sectional area, APW anterior-posterior width, LRW left-right width, LTSS light touch sensory scores, PPSS pinprick sensory scores, ISNCSCI International Standard for the Neurological Classification of Spinal Cord Injury.

The CSA, APW, and LRW in the AIS A/B group were significantly lower than those in the TD group (CSA: *p* = 0.002; APW: *p* = 0.001; LRW: *p* = 0.029), while CSA and LRW were significantly smaller than AIS C/D group (CSA: *p* = 0.014; LRW: *p* = 0.026). However, no statistical differences were found between the AIS C/D group and the TD group (Table 2, Fig. 2). For children with TLSCI, the CSA was positively correlated with PPSS (*r* = 0.430, *p* = 0.018). APW (Fig. 3)was positively correlated with PPSS (*r* = 0.470, *p* = 0.009) and LTSS (*r* = 0.382, *p* = 0.037). Neither sensory nor motor scores were associated with LRW.

**Table 2:** Morphologic spinal cord parameters at C2/3 level.

	TD mean (SD), range	AIS A/B mean (SD), range	AIS C/D mean (SD), range
CSA (mm^2^)	57.34 (4.39), 50.73–63.54	51.28 (5.17), 43.74–62.03	55.66 (3.93), 49.91–63.48
APW (mm)	6.90 (0.31), 6.52–7.46	6.41 (0.37), 5.76–7.03	6.68 (0.37), 6.16–7.23
LRW (mm)	10.49 (0.57), 9.58–11.41	9.97 (0.67), 8.77–11.15	10.48 (0.47), 9.77–11.49

*TD* typically developing, *AIS* American Spinal Injury Association Impairment Scale, *CSA* cross-sectional area, *APW* anterior-posterior width, *LRW* left-right width.

**Fig. 4:**
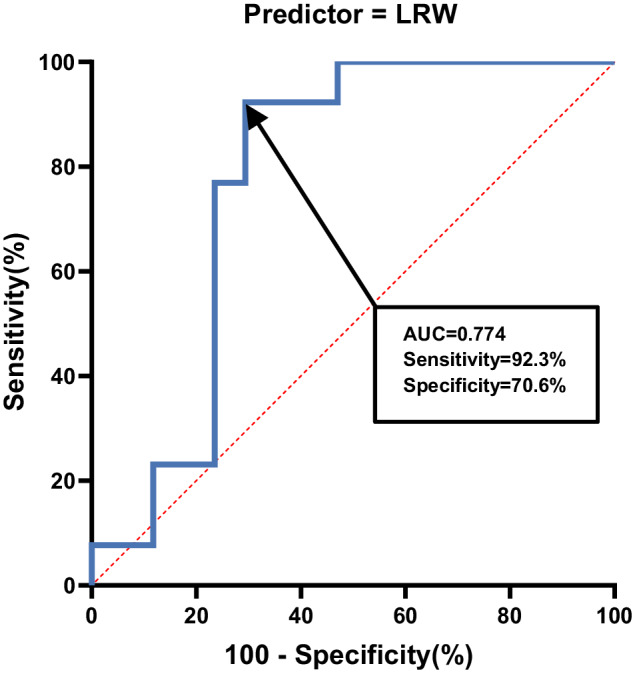
Receiver operating characteristic (ROC) curve between AIS A/B group and AIS C/D group, when using the LRW as the predictor. The area under the curve (AUC) for the ROC was 0.774 with a sensitivity of 92.3% and a specificity of 70.6%. LRW left-right width, AUC area under the curve, ROC receiver operator characteristic, AIS American Spinal Injury Association Impairment Scale.

In children with TLSCI, the results of ROC analysis for morphologic spinal cord parameters in AIS A/B and AIS C/D groups are shown in Table 3. Figure 4 shows the sensitivity and specificity of LRW, with a sensitivity of 92.3%, a specificity of 70.6%, and an area under the curve (AUC) of 0.77 (95% CI, 0.594–0.952).

**Table 3:** Results of receiver operator characteristic (ROC) curve analysis of the morphologic spinal cord parameters in children with TLSCI.

Test result variable(s)	AUC	Sensitivity	Specificity	Cut-off Value	Std. error^a^	Asymptotic Sig.^b^	Asymptotic 95% confidence interval	
							Lower bound	Upper bound
CSA	0.749	100%	47.1%	49.76	0.086	0.013	0.400	0.939
LRW	0.774	92.3%	70.6%	10.09	0.091	0.011	0.594	0.952
Combination (CSA + LRW)	0.765	52.9%	100%	0.72	0.087	0.014	0.594	0.936

*AUC* area under the curve, *ROC* receiver operator characteristic, *TLSCI* thoracolumbar spinal cord injury, *CSA* cross-sectional area, *LRW* left-right width.

^a^Under the nonparametric assumption.

^b^Null hypothesis: true area = 0.5.

### Multiple linear regression

The impact of age at inclusion, time since injury (months), and clinical grading at inclusion on CSA, APW, and LRW were investigated using multiple linear regression. Referring to the previous article, scoring of the extent of the clinical grading was done based on the AIS grades-A five points system (4–0; AIS A to AIS E in order of decreasing sensorimotor deficit) [22, 23].

Multiple linear regression analysis was performed on CSA, and the multiple linear regression model was statistically significant (*F* = 10.812, *P* < 0.001, R^2^ = 0.555). The regression equation: CSA = 56.473 mm^2^ + 0.682 (age) − 0.157 (time since injury) − 2.147 (clinical grading). Where age (β = 0.299, *p* = 0.038) significantly and positively influenced CSA. Time since injury (β = −0.525, *p* = 0.001) and AIS grade (β = −0.549, *p* < 0.001) significantly and negatively influenced CSA.

When multiple linear regression analysis was performed on APW, it was found that the model was statistically significant (*F* = 9.068, *P* < 0.001, R^2^ = 0.511). The regression equation: APW = 6.717 mm + 0.051 (age) − 0.014 (time since injury) − 0.127 (clinical grading). Where age (β = 0.30, *p* < 0.047) significantly and positively affected APW, time since injury (β = −0.014, *p* < 0.001), and clinical grading (β = −0.431, *p* = 0.004) significantly and negatively affected APW.

Multiple linear regression analysis was performed on LRW, and the multiple linear regression model was statistically significant (*F* = 3.253, *P* = 0.038, R^2^ = 0.273). The regression equation: LRW = 10.624 mm + 0.043 (age) − 0.007 (time since injury) − 0.238 (clinical grading). Where only clinical grading (β = −0.489, *p* = 0.007) significantly and negatively affected LRW.

## Discussion

This study focused on structural changes in the cervical spinal cord in children with TLSCI. The results of this study demonstrated that spinal cord atrophy (C2/3 level) occurred in children with TLSCI. In children with TLSCI, APW and CSA correlated with sensory scores, whereas LRW can be used as an imaging biomarker to distinguish whether the motor function is preserved below the neurological level of injury (NLI) in children with SCI. Therefore, morphologic spinal cord parameters in children with SCI can be used as objective imaging biomarkers to help reflect the functional status of children with SCI.

Previous studies have confirmed degenerative changes in the distal spinal cord after SCI, leading to spinal cord atrophy [5, 6, 24]. However, most of the previous studies focused on adult SCI. Unlike adults, the area of the spinal cord in TD children increases with age [18, 19]. To reduce the possible effect of age on the study, we ensured that there was no statistically significant difference in the mean age of the participants in the three groups. Compared to the TD group, the AIS A/B group had significantly reduced CSA, APW, and LRW parameters. The reduction in CSA and LRW might reflect retrograde changes in the corticospinal tracts after SCI, whereas APW might reflect Wallerian degeneration in the posterior columns of the spinal cord [25]. However, there were no significant differences in CSA, APW, or LRW between the TD group and the AIS C/D group. The mean values of the three indicators in the AIS C/D group were smaller than those in the TD group, which may also indicate to some extent that atrophy of the spinal cord occurred in the AIS C/D group but was not significant. Compared with the AIS C/D group, CSA and LRW in the AIS A/B group were lower than those in the AIS C/D group. This may indicate that spinal cord atrophy after SCI in children is related to clinical grading. The more severe the injury, the greater the reduction in spinal cord area.

The spinal cord serves as the primary conduit for the transmission of motor and sensory information between the brain and body [22] Sensory pathways are mainly located in the dorsal column of the spinal white matter, whereas motor pathways are mainly located in the lateral column [14]. It has been confirmed that APW and LRW can be used to independently assess sensory and motor function in adults with SCI [11]. In this study, the APW in children with TLSCI correlated with the two ISNCSCI sensory scores (Fig. 2). APW can reflect sensory function in children with TLSCI. Some children in the AIS A/B group retain some sensory functions [11, 22]. This may be the reason why there is no statistically significant difference in APW between the AIS A/B and AIS C/D groups. However, no correlation was found between LRW and ISNCSCI motor scores. This may be related to the poor precision of ISNCSCI motor scores in children under 15 years old [15]. The results of the ROC analysis showed that LRW was the best indicator to distinguish between the AIS A/B group and the AIS C/D group. The most important difference between the AIS A/B group and the AIS C/D group in children with TLSCI is whether there was the preservation of motor function below the NLI [22]. This suggested that LRW may reflect the preservation of motor function below the NLI in children with TLSCI.

For TD children, the spinal cord at the C2/C3 level grows faster with age than at other vertebral levels [18]. However, the relationship between the age of children with SCI and the spinal cord structure at the C2/3 level remains unclear. Factors such as age, time since injury, and clinical grading at inclusion may all potentially influence the structure of the cervical spinal cord. Therefore, we used multiple linear regression analysis in our study. The results of this analysis revealed that age was significantly positively correlated with both the CSA and the APW. Conversely, clinical grading at inclusion and time since injury were found to significantly negatively impact these measures. For the LRW, only clinical grading had a significant negative effect. These findings suggest that the spinal cord of children with SCI continues to develop with age, indicating that the effect of age may need to be considered in future studies. CSA was also found to progressively atrophy over time since injury, a finding consistent with previous studies [5, 8]. Research on SCI in adults has revealed a strong correlation between slower longitudinal changes in the spinal cord after SCI and better clinical outcomes [10, 25]. To our knowledge, longitudinal changes in the spinal cord after SCI in children have not been studied. This will be the focus of our future research, aiming to understand the longitudinal changes in spinal cord structure after SCI in children and to identify relationships with clinical outcomes to aid in predicting neurological outcomes. By monitoring neurodegenerative changes over time in children with SCI, we can assess the effectiveness of existing treatments and the potential effectiveness of new treatments to guide clinical practice and drug development. Notably, according to the multiple linear regression model, only the clinical grading was significantly negatively correlated with LRW. This suggests that LRW could serve as a relatively stable imaging biomarker for the long-term follow-up of children with SCI.

The ISNCSCI is the most commonly used for classifying SCI in adults and children and has been the main predictor of neurological function recovery [15, 16]. However, ISNCSCI may not accurately assess neurological function in young children with SCI [4]. This study found that the structural changes of the cervical spinal cord could reflect the motor and sensory functions of children with TLSCI. With the development of semi-automated and automated approaches to study spinal cord structures, morphologic spinal cord parameters of the cervical spinal cord may be objective imaging biomarkers for evaluating neurological function in children with SCI, and provide a simple and reproducible method for assessing neurological function in children with SCI.

## Limitations

Several limitations warrant consideration in this study. Firstly, our sample size was relatively small, and all children with TLSCI. Secondly, this study used a cross-sectional design, which could not reveal long-term evolutionary changes in the cervical spinal cord after SCI. Finally, although we recruited male participants, they were not included in this study due to the small number of participants. In China, the main cause of pediatric SCI is hyperextension of the spine during dance, resulting in a higher proportion of females with pediatric SCI [26]. And gender is a factor that significantly influences the spinal cord area [19]. To exclude the impact of gender, only females were included in our study.

## Conclusion

Quantitative measurement of morphologic parameters of the cervical spinal cord may be used as objective imaging markers to assess the motor and sensory functions of children with SCI. Atrophy of the cervical spinal cord (C2/3 level) in children with TLSCI, and that spinal cord atrophy was related to clinical grading. APW and CSA can reflect the sensory function in children with SCI; LRW may be an objective and stable imaging biomarker for evaluating motor function in children with SCI.

## Data Availability

The datasets generated and/or analyzed during the current study are available from the corresponding authors on request.
